# Mesoangioblasts at 20: From the embryonic aorta to the patient bed

**DOI:** 10.3389/fgene.2022.1056114

**Published:** 2023-01-04

**Authors:** Giulio Cossu, Rossana Tonlorenzi, Silvia Brunelli, Maurilio Sampaolesi, Graziella Messina, Emanuele Azzoni, Sara Benedetti, Stefano Biressi, Chiara Bonfanti, Laricia Bragg, Jordi Camps, Ornella Cappellari, Marco Cassano, Fabio Ciceri, Marcello Coletta, Diego Covarello, Stefania Crippa, M. Gabriella Cusella-De Angelis, Luciana De Angelis, Arianna Dellavalle, Jordi Diaz-Manera, Irene Fancello, Daniela Galli, Francesco Galli, Cesare Gargioli, Mattia F. M. Gerli, Giorgia Giacomazzi, Beatriz G. Galvez, Hidetoshi Hoshiya, Maria Guttinger, Anna Innocenzi, M. Giulia Minasi, Laura Perani, Stefano C. Previtali, Mattia Quattrocelli, Martina Ragazzi, Urmas Roostalu, Giuliana Rossi, Sabrina Santoleri, Raffaella Scardigli, Dario Sirabella, Francesco Saverio Tedesco, Yvan Torrente, Gonzalo Ugarte

**Affiliations:** ^1^ Division of Cell Matrix Biology and Regenerative Medicine, University of Manchester, Manchester, United Kingdom; ^2^ Division of Neuroscience, IRCCS Ospedale San Raffaele, Milan, Italy; ^3^ Muscle Research Unit, Charité Medical Faculty and Max Delbrück Center, Berlin, Germany; ^4^ School of Medicine and Surgery, University of Milano Bicocca, Milan, Italy; ^5^ Translational Cardiomyology Laboratory, Stem Cell and Developmental Biology Unit, Department of Development and Regeneration, KU Leuven, Leuven, Belgium; ^6^ Histology and Medical Embryology Unit, Department of Anatomy, Forensic Medicine and Orthopaedics, Sapienza University, Rome, Italy; ^7^ Department of Biosciences, University of Milan, Milan, Italy; ^8^ UCL Great Ormond Street Institute of Child Health and NIHR GOSH Biomedical Research Centre, London, United Kingdom; ^9^ Department of Cellular, Computational and Integrative Biology (CIBIO) and Dulbecco Telethon Institute, University of Trento, Trento, Italy; ^10^ Bayer AG, Research and Development, Pharmaceuticals, Berlin, Germany; ^11^ Department of Pharmacy-Drug Sciences, University of Bari “Aldo Moro”, Bari, Italy; ^12^ Lunaphore Technologies SA, Tolochenaz, Switzerland; ^13^ AGC Biologicals, Milan, Italy; ^14^ San Raffaele-Telethon Institute of Gene Theray, IRCCS Ospedale San Raffaele, Milan, Italy; ^15^ Department of Anatomy, University of Pavia, Pavia, Italy; ^16^ Cord Blood Bank, InScientiaFides, San Marino; ^17^ John Walton Muscular Dystrophy Research Centre, Newcastle University, United Kingdom; ^18^ Department of Medicine and Surgery, University of Parma, Parma, Italy; ^19^ Department of Biology, University of Tor Vergata, Rome, Italy; ^20^ UCL Department of Surgical Biotechnology and Great Ormond Street Institute of Child Health, London, United Kingdom; ^21^ CellCarta, Gosselies, Belgium; ^22^ Department of Biochemistry and Molecular Biology, Faculty of Pharmacy, Universidad Complutense de Madrid, Madrid, Spain; ^23^ CellFiber Co., Ltd, Tokyo, Japan; ^24^ IFO, Istituti Fisioterapici Ospedalieri, Rome, Italy; ^25^ Lavitaminasi, Clinical Nutrition and Reproductive Medicine, Rome, Italy; ^26^ Division of Molecular Cardiovascular Biology, University of Cincinnati, Cincinnati, OH, United States; ^27^ Gubra ApS, Horsholm, Denmark; ^28^ Roche Institute for Translational Bioengineering (ITB), pRED Basel, Basel, Switzerland; ^29^ Institute of Translational Pharmacology, National Research Council, Rome, Italy; ^30^ Columbia Stem Cell Initiative, Department of Rehabilitation and Regenerative Medicine, Columbia University, New York, United States; ^31^ University College London, Great Ormond Street Hospital for Children and the Francis Crick Institute, London, United Kingdom; ^32^ University College London, Great Ormond Street Hospital for Children and The Francis Crick Institute, London, United Kingdom; ^33^ Neurology Unit, Fondazione IRCCS Ca’ Granda Ospedale Maggiore Policlinico, Milan, Italy; ^34^ Stem Cell Laboratory, Department of Pathophysiology and Transplantation, Dino Ferrari Center, University of Milan, Milan, Italy; ^35^ Laboratory of Neuroscience, Faculty of Chemistry and Biology, University of Santiago de Chile, Santiago, Chile

**Keywords:** mesoderm, myogenic stem cells, pericyte, muscular dystrophy, muscle development

## Abstract

In 2002 we published an article describing a population of vessel-associated progenitors that we termed mesoangioblasts (MABs). During the past decade evidence had accumulated that during muscle development and regeneration things may be more complex than a simple sequence of binary choices (e.g., dorsal vs. ventral somite). LacZ expressing fibroblasts could fuse with unlabelled myoblasts but not among themselves or with other cell types. Bone marrow derived, circulating progenitors were able to participate in muscle regeneration, though in very small percentage. Searching for the embryonic origin of these progenitors, we identified them as originating at least in part from the embryonic aorta and, at later stages, from the microvasculature of skeletal muscle. While continuing to investigate origin and fate of MABs, the fact that they could be expanded *in vitro* (also from human muscle) and cross the vessel wall, suggested a protocol for the cell therapy of muscular dystrophies. We tested this protocol in mice and dogs before proceeding to the first clinical trial on Duchenne Muscular Dystrophy patients that showed safety but minimal efficacy. In the last years, we have worked to overcome the problem of low engraftment and tried to understand their role as auxiliary myogenic progenitors during development and regeneration.

## The developmental origin of myogenic and osteogenic cells

In vertebrates, skeletal myoblasts originate from dorsal somites in the trunk and from presomitic paraxial mesoderm in the head. Bones rather originate from ventral somite in the trunk, lateral mesoderm in the limbs and neural crest in the head. This notion came from pioneering chick-quail transplantation studies (Couly et al., 1993; Monsoro-Burq et al., 1994; Chevallier et al., 1977; Christ et al., 1977).

Skeletal muscle development starts right after the onset of somitogenesis and is completed at the end of post-natal growth (Buckingham 2017), though adult muscle maintains the ability to regenerate after injury. Therefore, it was safe to postulate the existence of different progenitor cells that could undergo different fates (differentiation versus proliferation) in the same micro-environment to accommodate the need of providing early motility and structure to the embryo while increasing the mass of the tissue (Cossu and Molinaro, 1987). In fact, muscle and bone cells stop dividing when differentiated, therefore new cells must be added by fusion to the growing fibres or apposition to the growing bone. Canonical progenitors, active throughout growth, are osteoblasts underneath the endosteum layer (Bianco 2014) and satellite cells underneath the endomysium (Engquist and Zammit, 2021). However, it has been shown that bone pericytes in trabecular bone have the ability to give rise to new osteocytes (Bianco 2014) and we showed that pericytes of skeletal muscle contribute to post-natal development of multinucleated muscle fibres (Dellavalle et al., 2011).

## How MABs were identified

In the mid 90’ the first transgenic mice expressing the LacZ reporter were generated, thanks to the work of Jean Francois Nicholas (Sanes et al., 1986). For the first time a long-lasting dream of mammalian embryologists came true: a cell autonomous, eventually tissue specific, inheritable marker that would allow following the fate of specific cell types (e.g. myogenic) *in vivo*. The senior author of this review had imported to his lab, then in Rome, MLC1/3F-nLacZ mice (Kelly et al., 1995) from Margaret Buckingham lab in Pasteur. All and only striated muscle cells in these mice express the LacZ gene with a nuclear localization signal. When we co-cultured unlabelled C2C12 myoblasts with LacZ + fibroblasts, we found LacZ + myotubes, easily identified by a nuclear blue staining with X-Gal that reveals the activity of LacZ-encoded β-galactosidase. LacZ + fibroblasts, cultured alone or with other cells, did not show any staining. We concluded that fibroblasts, as then we would define any non-myogenic cell in skeletal muscle (for an overview of muscle interstistial cells, see Table 1) did not possess myogenic potential on their own, but may be recruited to myogenesis by adjacent differentiating myogenic cells, either by fusion or by short-range paracrine signals (conditioned medium did not work) that may render fibroblasts competent to fuse. We published this paper in 1995 (Salvatori et al., 1995), and almost simultaneously two similar papers by the groups of Diane Watt and Louis Garcia (Breton et al., 1995; Gibson et al., 1995) reported very similar data, in agreement with our observation. As it always happens with reports that disagree with current dogmas, the papers were simply ignored or dismissed as “tissue culture artefacts”. Thus, we decided to test whether the phenomenon might also occur *in vivo* without any intermediate culture. So, we transplanted bone marrow from MLC1/3F-nLacZ mice into wt adult animals. We observed that, following an acute injury to muscle but not under basal conditions, nuclei expressing LacZ were detected in regenerated muscle fibres (Ferrari et al., 1998). The frequency was low but reproducible in all mice and reproduced in other laboratories who tried to use bone marrow derived cells to repair muscle in dystrophic mice (Gussoni et al., 1999), though the frequency was too low to justify any realistic hope. Our paper had an impact (cited more than 2.400 times so far) and opened the controversial field of cell plasticity. Within months many publications appeared in high profile journals, often supported only by few low-resolution immune-fluorescence analysis, showing that virtually any stem/progenitor cell of our body may give rise to any differentiated progeny. This led to premature clinical translation, especially for heart diseases, eventually to be repaired with bone marrow derived cells (Chien et al., 2019). Overall, the field was mudded and results dismissed as technical tissue culture artefacts (Bonfanti et al., 2012).

**TABLE 1:** Adult stem/progenitor cells involved in skeletal muscle regeneration, beside satellite cells and other muscle derived myogenic cells; MABs, mesoangioblasts; PICs, PW1(+)/Pax7 (-) interstitial cells; SP, side population; FAPs, Fibro-adipogenic progenitors; HSCs, hematopoietic stemcells; MSCs, Bone marrow stromal cells; MiPs mesodermal iPSC-derived progenitors; SMCs, smooth muscle cells.

Cell type	Identification markers		Differentiation potential (mainly)		References
	*Positive*	*Negative*	*in vitro*	*in vivo*	
*Mesoangioblasts (MABs)	AP, NG2, cKit, Flk-1, Sca-1, CD44, CD140, CD146	CD45, CD34, CD56, CD144, Pax7	Myogenic Adipogenic SMCs	MyogenicSMCs	Minasi et al., 2002
Interstitial cells (PICs)	PW-1, CD34, Sca-1	Pax7, Pax3			Mitchell et al., 2010
Side population (SPs)	Sca-1/ABCG2, Syndecan4, Pax7	CD45, CD43, cKit, CD11, Gr-1, B220, CD4, CD8	Myogenic	Myogenic	Tanaka et al., 2009
Skeletal muscle- CD34+/45- (Sk-34)	CD34	CD45, Pax7, CD73	Myogenic	Myogenic	Tamaki et al., 2007
*Fibro-adipogenic progenitors (FAPs)	CD34, Sca-1, CD140a CD73	CD45, Lin, CD31, α-7 integrin	Adipogenic Fibrogenic	Adipogenic Fibrogenic	Joe et al., 2010
hematopoietic stem cells (HSCs)	CD45, cKit, Sca-1, CD34	Lin	HematopietiMyogeni	lineages Myogenic	Ferrari et al., 1998
*Bone marrow stromal cells (MSCs)	CD29, CD44, CD90, CD76, CD166, CD106, CD71, CD73, CD105	CD45, CD34, CD14, CD133	Myogenic	Myogenic	Dezawa et al., 2005
*iPSC-mesodermal progenitors (MiPs)	CD140a, CD140b, and CD44		Mesodermal lineages	Mesodermal lineages	Quattrocelli et al., 2015

*These cells have been described also from human samples.

**FIGURE 1:**
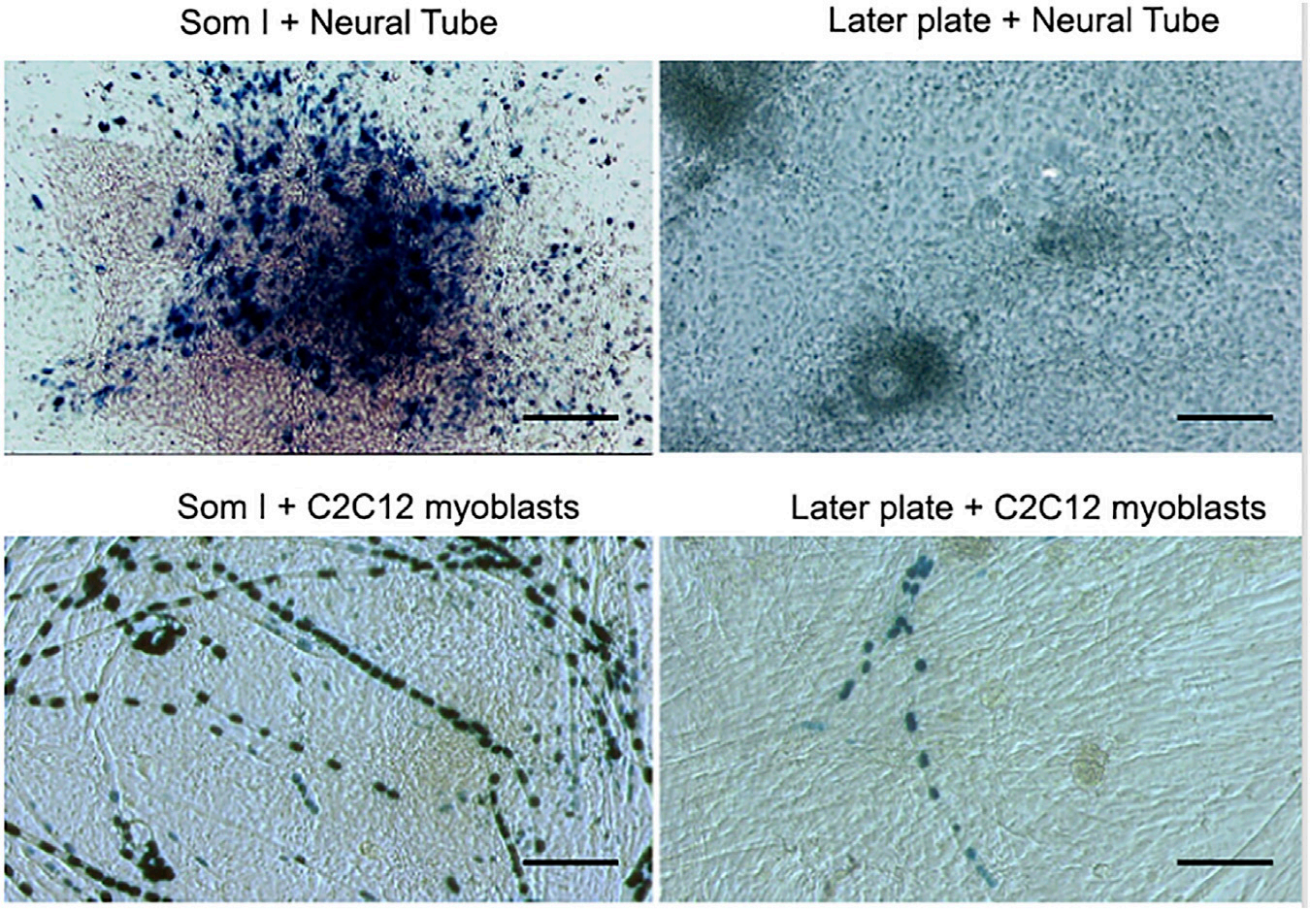
A co-culture of paraxial (Som I) mesoderm or Lateral Plate from MLC1/3F-nLacZ embryos with either neural tube from wt embryos or with unlabelled C2C12 myoblasts. After 5 days, cultures were fixed and stained with X-Gal.

In the meanwhile, we decided to investigate where these progenitors originate from during mouse development. In the absence of any specific marker, we isolated and cultured as tissue explants somites, lateral plates, neural tube, heart, and aorta. After a week, we cloned embryonic cells outgrown from the explants and observed that clones from dorsal aorta had the clear morphology of satellite cells and co-expressed early endothelial and myogenic markers such as MyoD. Of notice the culture of the same undissociated explant did not differentiate into skeletal muscle cells. Years later, we showed that explants of embryonic aorta from MLC1/3F-nLacZ mice, when cultured on C2C12 or primary wt myoblasts, would give rise to LacZ + nuclei in hybrid myotubes. This process was stimulated by Noggin and inhibited by BMP (Ugarte et al., 2012). Moreover, when lateral plate from these embryos at early somite stage (E 8.5) was isolated and cultured onto C2C12 myogenic cells, several myotubes containing LacZ + blue nuclei could be observed, similarly, though at a lesser extent, than when paraxial mesoderm was co-cultured with the same C2C12 cells. In contrast, when lateral plates or paraxial mesoderm were cultured with wt neural tubes, only paraxial mesoderm gave rise to very many LacZ + mononucleated myocytes, while lateral plate did not (Figure 1). This result showed that the lateral plate, from which the vasculature originates, contains cells that cannot be induced to skeletal myogenesis by the neural tube as the newly formed somites (Vivarelli and Cossu, 1986) but can be “recruited” to myogenesis by differentiating muscle cells.

This led us to believe that an early endothelial progenitor may be endowed with skeletal myogenic potency, to be revealed once the cells were exposed to conditions promoting skeletal myogenesis (De Angelis et al., 1999). We confirmed these data with quail-chick transplantation experiments where quail, but also mouse dorsal aorta was transplanted into an early chick limb bud: cells from the transplanted aorta gave rise to most mesodermal tissues, including skeletal muscle (Minasi et al., 2002). When cells were isolated from the mouse aorta and cloned on feeder layers, they gave rise to clonal lines expressing both endothelial and pericyte markers that can proliferate indefinitely.

Transcriptomic analysis of these clones revealed a specific molecular signature, slightly overlapping with that of other stem cells but clearly different from embryonic fibroblasts (Tagliafico et al., 2004). The differentiation potential of these clonal lines was explored *in vitro* and in *vivo* assays. Dorsal aorta clones were able to promptly differentiate into smooth muscle cells in response to TGFβ1 and into osteoblast-like cells in response to BMP2, in accordance with their high level expression of many members of the TGFβ/BMP pathway. These cells also differentiated into adipocytes in response to insulin but were unable to undertake a chondrogenic pathway, under standard cartilage inducing conditions. Most importantly these cell lines would not differentiate spontaneously into skeletal muscle, unless co-cultured with myogenic cells or transplanted into regenerating skeletal muscle.

Because of their vascular markers and their ability to differentiate into several mesoderm tissues we named these cells “meso-angioblasts”, subsequently simplified into “mesoangioblasts” (MABs). Whether these MABs were a sub-population of endothelial progenitor cells, such as the EPC, described in (Gulati et al., 2003; Hur et al., 2004), or a subtype of pericytes (Cossu and Bianco, 2003), expressing also early endothelial markers remained unclear at the time.

## Possible origin of mesoangioblasts during embryogenesis

Endothelium had already been shown at the time to be a crossway of several cell lineages during embryogenesis. Fate-mapping and imaging studies of the developing dorsal aorta and work on embryonic stem cells, led to the discovery and characterization of the hemogenic endothelium, a specialized embryonic endothelial population that gives rise to the precursors of hematopoietic stem cells (HSCs) and therefore essentially to all the adult blood (Lancrin et al., 2009; Boisset, et al., 2010; Zovein, et al., 2010; Swiers et al., 2013).

A first strategy to investigate the endothelial embryonic origin of MABs in the dorsal aorta, was to adopt a genetic lineage tracing approach. To genetically label endothelial-derived cell populations, we used the Cdh5-CreERT2 transgenic mouse developed in the lab of Ralph Adams, that expresses a Tamoxifen (TAM)-inducible Cre recombinase (CRE-ERT2) under the control of VE-Cadherin (VE-Cad) regulatory sequences (Wang, et al., 2010). By inducing the expression of the Cre recombinase at E8.5 in a double transgenic Cdh5-CreERT2; R26R-EYFP, we were able to permanently label a population of progenitors with mesodermal potency, that originates from VE-Cad + endothelial cells within the yolk sac and in the placenta and are found later in the embryo proper (Azzoni et al., 2014). These VE-cad + derived cells, expressing hematopoietic (CD45^+^) but not myeloid/macrophage markers (CD11b-), displayed, in the developing embryos, a tissue distribution overlapping with the one observed with transplantation of MABs cell lines (smooth and skeletal muscle, dermis).

**FIGURE 2:**
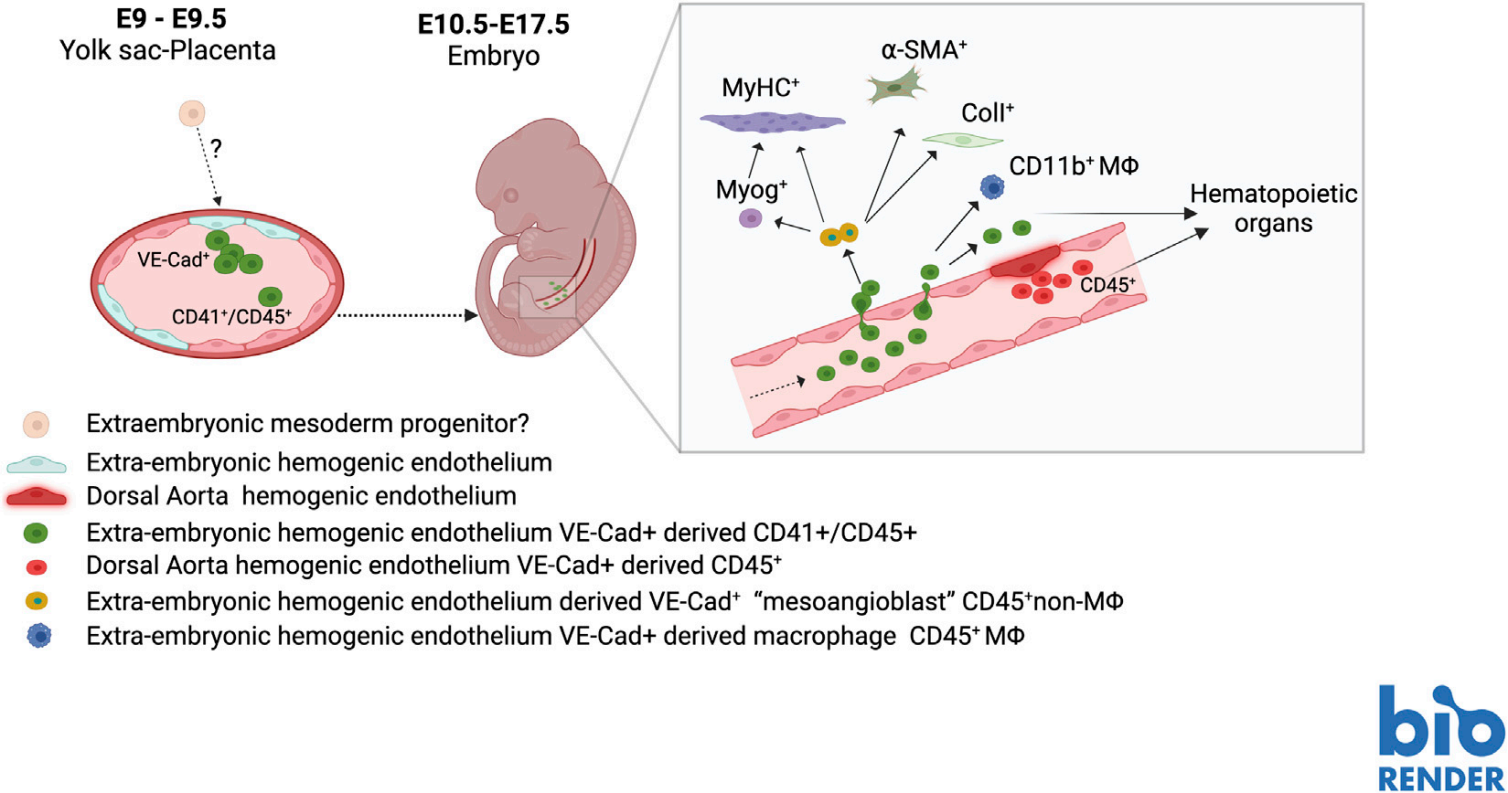
Hypothetical scheme of the origin of MABs in during mouse embryo development.

Most interestingly, isolated VE-Cad + -derived CD45^+^ CD11b-cells displayed MABs features in *vitro* and *in vivo* assays. They could indeed differentiate in osteoblasts and smooth muscle when treated with BMP or TGFβ respectively and could give rise to MyHC + myotubes in co-culture with myoblasts. In addition, they were able to contribute to post-natal muscle regeneration *in vivo* when transplanted in the muscle of wt or dystrophic mice. However, like the dorsal aorta cell lines, VE-Cad + -derived CD45^+^ CD11b-cells were unable to spontaneously differentiate in skeletal muscle *in vitro*, and no VE-cad-derived cells could be detected in skeletal muscle after birth (Azzoni et al., 2014). All this evidence suggested that embryonic MABs are a transient sub-population of hemogenic endothelium-derived cells endowed with a mesoderm multi-lineage differentiation potential that is restricted to the embryonic and foetal normal development but is not maintained in postnatal life (Figure 2).

## Post-natal MABs originate from muscle pericytes: fate and potency

Indeed, when we isolated what we thought were the same progenitors from post-natal murine and human muscle, we noticed that they only expressed pericyte and not endothelial markers. Moreover, they would tend to differentiate spontaneously into smooth muscle (the default pathway of pericytes) and skeletal muscle but no other mesoderm tissues, suggesting a restriction of potency concomitant with a tissue-specific commitment. Indeed, many years later, Paolo Bianco and others showed that there are no identical mesenchymal stem cells: pericytes isolated from human bone would differentiate, using rigorous assays, only into osteocytes, chondrocytes and adipocytes, the components of bone, beside smooth muscle. Conversely, pericytes isolated from human skeletal muscle would differentiate into skeletal and smooth muscles, as stated above (Sacchetti et al., 2016).

A possible interpretation of these data would postulate that vessel associated progenitors may contribute to the post-embryonic growth of tissues. In mammals all organs are formed during embryogenesis at the end of which, a whale, a man and a mouse have very similar size (Roston et al., 2013). Thus, most of the body is built during foetal and post-natal growth by cell division, mainly for epithelia, or by cell addition mainly for solid mesoderm such as bone and skeletal muscles, where differentiated cells no longer divide (Bianco and Cossu, 1999).

**FIGURE 3:**
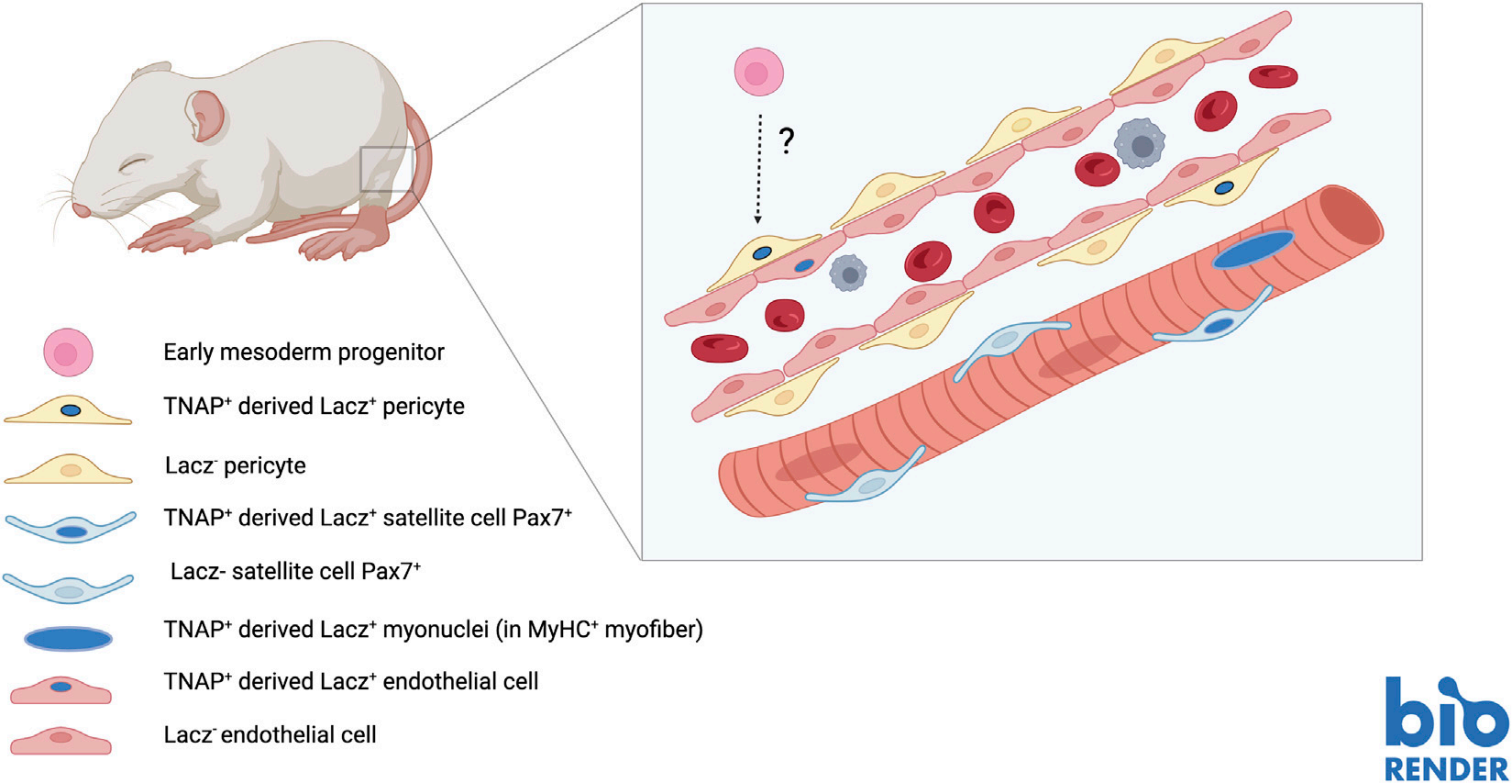
Contribution of TNAP + pericyte (MABs) to developing foetal muscle and satellite cells.

To carry on a lineage tracing analysis of vessel-associated postnatal progenitors and assess their *in vivo* contribution to the growth of the skeletal muscle, we needed a specific marker. Microarray analysis of *in vitro* expanded MAB cell populations from post-natal and juvenile muscle showed the expression of pericyte markers (e.g., NG2, MCAM and PDGF-Rb) which almost invariably are also expressed, though at low level, by skeletal myogenic progenitors (Tagliafico et al., 2004; Dellavalle et al., 2007). In other words, there is not a unique marker, such as “MyoD” for skeletal myogenic cells, that uniquely identifying these progenitors. Tissue Non-specific Alkaline Phosphatase (TNAP) was the only marker we found not shared with myoblasts. However, as all pericyte markers, also TNAP is expressed on many other cell types, including endothelium, and is not expressed in all pericytes. This prevented a prospective isolation of MABs and confined lineage tracing studies to the use of a TNAP-Cre^ERT^ mouse that we generated in collaboration with Sharaghim Tajbakhsh (Dellavalle et al., 2011). In addition, we could not lineage trace TNAP + pericytes before birth because TNAP is expressed by many other cell types, whereas postnatally its expression is restricted to pericytes in humans and to pericytes and endothelium in rodents (Safadi et al., 1991). By administering Tamoxifen to P6 pups, we observed labelling of small vessels only, as predicted. However, after 1 month many muscle fibres were LacZ+, indicating that some cell previously expressing TNAP had fused with growing muscle fibres. We also noticed that contribution varied from almost 10% of positive fibres (i.e., containing nuclei derived from TNAP + pericytes) in the diaphragm to less than 1% in the Tibialis anterior. This is interesting in light of subsequent observations showing a higher contribution of satellite cells to these muscles in comparison with hind limb muscle (Keefe et al., 2015; Pawlikowski et al., 2015). Indeed we detected also LacZ + satellite cells, indicating a Mabs contribution to the satellite cell pool, but this was too low to account for the reported difference in satellite cell abundance among these different muscles. Following acute injury or in a dystrophic background, the percentage of positive fibres increased in proportion to the labelling observed in healthy muscle. Most importantly the contribution of pericytes to skeletal muscle could be observed only during post-natal growth. When adult animals (P30 or older) were treated with Tamoxifen, the contribution of TNAP + cells to muscle fibres was negligible. And this also explains the apparent discrepancy with a subsequent paper, claiming that TBX18 + pericytes do not contribute to any non-vascular tissue (Guimarães-Camboa et al., 2017). This work was based on Tamoxifen administration at P30, so that contribution to skeletal muscle could not be observed because it occurs, at least for TNAP + pericytes, only during post-natal growth (Figure 3).

These results are in agreement with our hypothesis that a vessel-associated progenitor, most likely an adventitial pericyte (TNAP+), may generate cells fated to differentiate into one or another type of solid mesoderm, depending upon the tissue where the vessel would ingress during foetal angiogenesis, thus being recruited by local differentiating cells (Cossu and Bianco, 2003). Whether these postnatal TNAP + vessel progenitors have a common mesodermal ancestor with the embryonic hemogenic endothelium derived MABs is still unknown, due to the lack of specific early markers for lineage tracing.

Regardless, we have been using the term “mesoangioblast”, MABs, to indicate not only the cultured cells derived from either embryonic endothelial progenitors and pericytes in the post-natal muscle, but also their *in vivo* counterparts, even though this is not strictly correct as, for example, embryonic stem cells do not exist as such in the inner cell mass of the blastocysts but represent their *in vitro*-derived counterpart.

In the general context of the pericyte/myogenic cell relationship, the above data supported the idea that pericytes may differentiate into myogenic cells during post-natal muscle growth and this contribution may be enhanced by acute or chronic injury. However, pericytes alone were not sufficient to sustain muscle regeneration when all satellite cells were ablated, also considering that a possible by-stander effect (Bi et al., 1993): pericytes and satellite cells are often juxtaposed across the muscle basal lamina.

On the other hand, we observed that committed myogenic cells could be recruited to a pericyte fate by signals emanated by the endothelium such as Dll4 and PDGF-BB. Of notice both embryonic and foetal myoblasts (Cappellari et al., 2013) but also post-natal satellite cells (Gerli et al., 2019) could be converted to pericytes but maintained expression of Myf5, indicating that their myogenic identity had not been erased.

**FIGURE 4:**
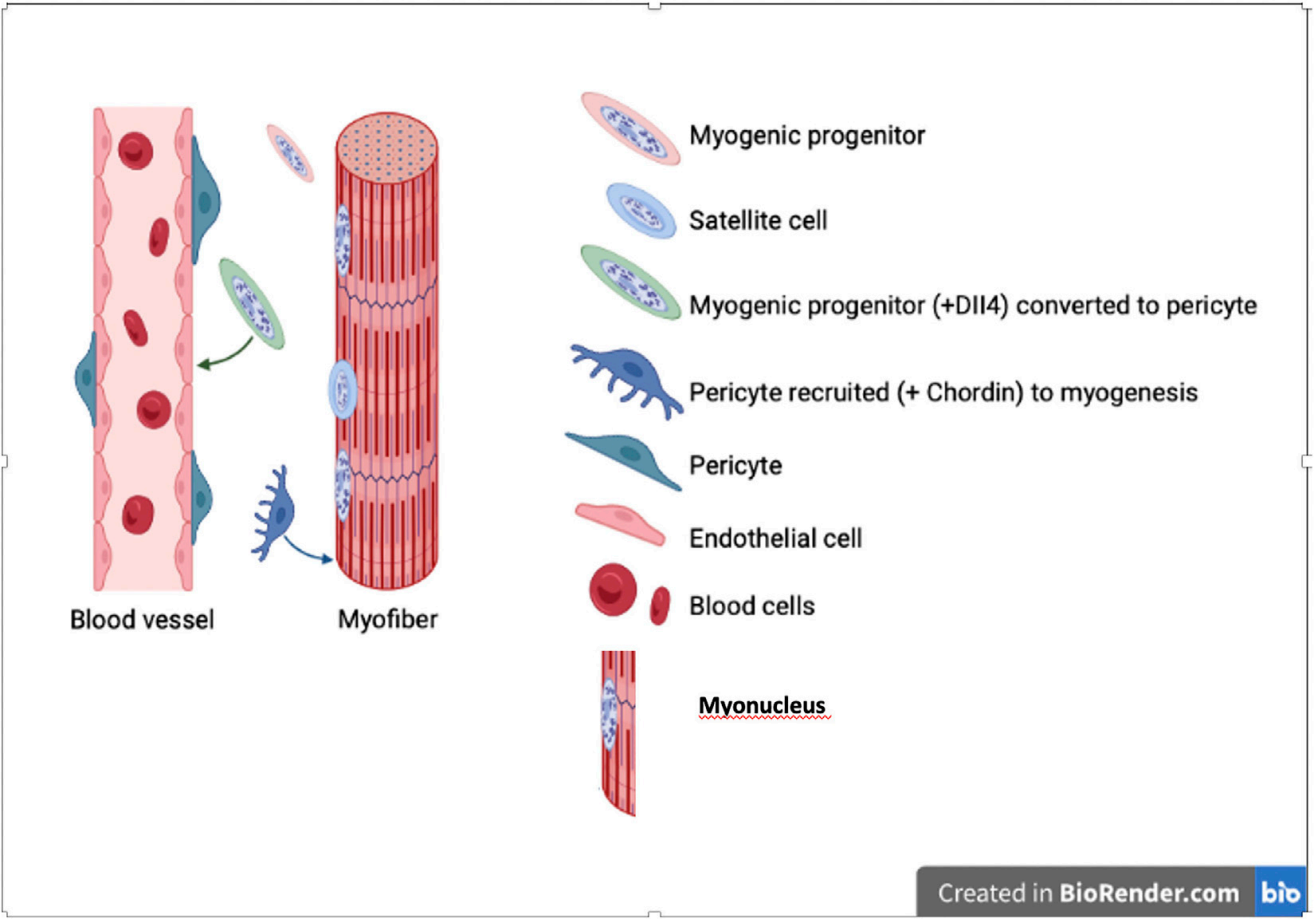
Bi-directional differentiation of myogenic cells into pericytes and *vice versa*. Myogenic cell nuclei are represented in salt and peper gray, while nuclei of non myogenic cells in uniform solid colour.

Thus, one may ask why there is this bi-directional differentiation potency and what role it may fulfil during development or in pathological situation (Stallcup, 2013). During development the equilibrium should usually be skewed towards skeletal muscle, overwhelming pericytes quantitatively, so that non myogenic cells may be recruited to increase the number of myogenic cells. However, when vessels grow into these developing or regenerating muscles, they may, in turn, recruit surrounding mesoderm cells, including myoblasts, to a pericyte fate, needed to stabilise the vessel (Figure 4)

## Mechanisms regulating MAB function and competence

The potential therapeutic applications of MABs for cell therapy of muscle disease made it particularly important to understand how their acquisition of a skeletal muscle fate is regulated.

Indeed, although the myogenic potential of MABs was progressively demonstrated *in vitro* and *in vivo*, the molecules regulating this process were still unknown. Genome-wide RNA expression analysis, performed on embryonic MABs, revealed a characteristic pattern of expression that correlates with the biological features and the developmental potency of these cells (Tagliafico et al., 2004). Interestingly, MABs were found to express Pax3 at a high level. The paired box/homeodomain transcription factors of the Pax family play important roles in the formation of tissues and organs during embryonic development, being implicated in neurogenesis in dorsal regions of the central nervous system, also by regulating neural crest and its derivatives, and playing a key role in skeletal myogenesis. In the latter context, Pax3 is expressed in paraxial mesoderm and marks multipotent cells of the dorsal somite, the dermomyotome, from which skeletal muscle is derived. Endothelial and smooth muscle cells are also derived from paraxial mesoderm, and the same Pax progenitor in the dermomyotome can give rise to both the smooth muscle cells of blood vessels and skeletal muscle (Bajard et al., 2006; Buckingham, 2017). We therefore generated embryonic MABs from *Pax3* null embryos (Messina et al., 2009). The absence of Pax3 in multipotent MABs did not affect their morphology, as also their typical gene expression profile required for their proliferation or survival as also their differentiation into the most mesodermal derivatives was not compromised. On the contrary, lack of Pax3 strongly impaired skeletal muscle differentiation, thus suggesting a crucial role of Pax3 in the commitment of embryonic mouse MABs to an inducible myogenic fate. Notably, Pax7 was dispensable, at least in the embryonic period. Consequently, MABs isolated from ^PAX3PAX3−FKHR-IRESnLacZ/+ ^mouse embryos, where the oncogenic fusion protein Pax3-FKHR rescues the early Pax3 mutant phenotype by over-activating Pax3 targets (Relaix et al., 1996), when cocultured with human satellite cells, differentiated into multinucleated myotubes with higher efficiency (20%–30% versus 5%–10%) than wt MABs. The fundamental role of Pax3 in MAB biology was also confirmed *in vivo* in *Sgca* null dystrophic animal with IA transplanted MABs (Messina et al., 2009). Therefore, our first study aimed at the identification of the molecular pathway(s) regulating MAB myogenic potential showed that Pax3, but not Pax7, regulates the differentiation of MABs into skeletal muscle, consistent with a possible paraxial mesoderm origin of these cells in the embryonic dorsal aorta (Esner et al., 2006; Messina et al., 2009).

However, signalling factors originating from differentiating muscle cells are necessary to induce embryonic MABs into skeletal myogenic programme, and their regulatory targets remain to be defined. Additionally, Pax3 seems not to be necessary for adult mouse MABs, as also for dog and human ones, supporting the hypothesis that embryonic and post-natal MABs are indeed similar but not identical cells.

In 2015, a cross-analysis of independent microarrays performed on different human and murine MAB populations revealed that PW1 is expressed in MABs regardless of species origin and age of isolation (Tagliafico et al., 2004; Bonfanti et al., 2015). PW1, also identified as Peg3 (Paternally expressed gene3), initially isolated following a screening to identify factors involved in skeletal muscle commitment as well as in a screen for parentally imprinted genes (Relaix et al., 2003), is expressed at high levels upon gastrulation and is downregulated during foetal and perinatal development (Kuroiwa et al., 1996). In postnatal skeletal muscle, PW1 expression is restricted to muscle satellite cells and to a subpopulation of interstitial cells (PICs, PW1-interstitial cells), which display myogenic potential (Mitchell et al., 2010; Pannerec et al., 2013). Moreover, PW1 expression identifies multiple adult stem and progenitor cell populations from a wide array of adult tissues including skin, gut, blood and central nervous system (Besson., 2011). We demonstrated that both mouse and human MABs displaying high levels of PW1 show a higher myogenic competence and ability to cross the vessel wall upon systemic delivery in dystrophic animal models. Indeed, lack of PW1 in MABs causes a block in myogenic differentiation, through a mechanism that drives MyoD to degradation *via* the proteasome machinery and a strong JAM-A dependent adhesion to the endothelium thus preventing MAB extravasation (Bonfanti et al., 2015). These studies from our team represented an important first step in the comprehension of MAB behaviour and competence. Indeed, among the multiple obstacles in using allogeneic cell transplantation for therapeutic ends, a key challenge is to identify the most appropriate donor cell, in addition to requirements related to the immune system such as HLA compatibility. At present, screening the competence of each preparation of MABs is time consuming and costly requiring both *in vitro* and ultimately *in vivo* assays. The striking correlation between the level of PW1 expression in MABs and their ability to differentiate into muscle and to cross the endothelium in numerous cell preparations across species and age suggests that assessing PW1 levels may represent a rapid and single step assay for screening donor cell preparations of therapeutic value.

## Mesoangioblasts, single cell OMICS and modelling muscle pathologies

Beside the satellite cells, canonical stem/progenitor cells of skeletal muscle, isolated more than 60 years ago (Mauro, 1961) and recently historically reviewed (Engquist and Zammit 2021), many stem/progenitor cell populations have been isolated and characterized from skeletal muscles in the last 20 years. Table 1, summarises and updates current knowledge on the currently characterized interstitial cells of skeletal muscle, previously known as “fibroblasts”. However, following the unbiased clustering by Smart-seq2 of interstitial cells from healthy and dystrophic mouse muscles, we were able to find distinct cell clusters only for muscle stem cells/satellite cells (MuSCs) and interstitial stem cells (ISCs). This was also observed by other groups who performed scRNA-seq on healthy skeletal muscle (Dell'Orso et al., 2019; Giordani et al., 2019). Within the ISC cluster, markers were found representing different ISC populations, including MABs, fibro-adipogenic progenitors (FAP) and PW1^+^/Pax7^–^ cells (PICs). However, the different adult stem cells (MABs, FAPs, PICs and so on) are present in the same cluster due to the similarity of single cell transcriptomic profiles, raising the issue of the reciprocal lineage relationship and possible inter-conversion among these stem/progenitors, as previously postulated (Bianco and Cossu, 1999). Indeed, ISCs have been defined by various isolation techniques and functions and have mostly only been compared with MuSCs to show their different origins. Nevertheless, despite these different isolation methods, ISCs share many overlapping markers. For example, FAP, PIC and TWIST2 + mouse progenitors are all positive for SCA-1 that was originally described in MABs (Dellavalle et al., 2007; Mitchell et al., 2010; Liu et al., 2017), which suggests that they may acquire this marker during culture conditions. MABs, PICs and TWIST2+ progenitors have been characterized for the ability to directly participate to adult myogenesis (Mitchell et al., 2010; Dellavalle et al., 2011; Liu et al., 2017) while FAPs only support indirectly adult myogenesis and mainly differentiate into adipocytes and myofibroblasts (Uezumi et al., 2010). Even so, it has been shown that MABs, PICs and TWIST2+ progenitors differentiate also into adipocytes, under different conditions. In addition, HDAC inhibitors can drastically impact FAP differentiation potential, driving them towards a myogenic fate (Saccone et al., 2014). These findings may have been due to different protocols adopted by different laboratories, also because cells were isolated from different muscles and/or different stages of different animal models. Despite that, when we isolated MABs and FAPs from wt and β-sarcoglycan KO mice and compared their cell potency, we observed a myogenic and an adipogenic capacity for both cell types (Camps et al., 2020). All these results highlight the importance of further studies using an unbiased comparison of all these cell populations with simultaneous cell isolation, large scRNA-seq screens, and functional assays *in vitro* and *in vivo* to shade light on adult muscle stem/progenitor cell heterogeneity.

Many studies have been directed to improve MAB proliferative capacity, myogenic potency and to overcome the problem of low engraftment; these investigated NOTCH (Quattrocelli et al., 2014) BMP (Costamagna et al., 2016) and PDGF (Camps et al., 2020) signaling, and we also tried to better understand their role during muscle development and regeneration (Quattrocelli et al., 2012; Giacomazzi et al., 2021; Ronzoni et al., 2021). It is clear that aging (Rotini et al., 2018) and the stem cell niche of cachectic muscles (Costamagna et al., 2020) negatively impact on the regenerative capacity of MABs and this should be carefully considered for their future potential therapeutic applications. A promising cell model for targeting muscle tissues is constituted by induced pluripotent stem (iPS) cells (Takahashi et al., 2007). At present, iPS cells can be obtained from a wide variety of somatic cells, including murine (Quattrocelli et al., 2015), equine (Quattrocelli et al., 2016) and human (Giacomazzi et al., 2017) MABs. Originally, iPS cells were reprogrammed from somatic cells by transiently overexpressing pluripotency regulators, such as OCT4, SOX2, KLF4 and cMYC, through retroviral vectors (Takahashi et al., 2007). iPS cells display many pluripotency features, including virtually unlimited proliferation potential and differentiation potency toward derivatives of all embryonic lineages including MAB-like cells (Tedesco et al., 2012). During reprogramming of somatic cells to a pluripotent state, tissue-specific genes and differentiation regulators must be silenced, while pluripotency genes and self-renewal genes must be activated. Hence the reprogramming of MABs to the pluripotent state includes genome-wide modulation of histones and DNA methylation that modify the epigenetic landscape offering, however, a differentiation bias towards myogenic lineages due to a persistent cell memory (Kim et al., 2010) that can be captured by small noncoding RNA signatures (Giacomazzi et al., 2017).

A bio-fabrication approach to restore skeletal muscle loss has been achieved using MABs (Costantini et al., 2021) and the latter are a valuable stem cell source to generate neuromuscular junction models in microfluidic devices (Stoklund Dittlau et al., 2021). These bidimensional cell systems are of great help to study cell-cell interactions and cell signalling and can be genetically manipulated (Santoni de Sio et al., 2008); however, more complex three-dimensional structures are necessary to better mimic the original muscle tissues and to contribute to the maturation of iPS cell derivatives. In fact, organoids as self-organized 3D structures, are composed of different types of cells derived from stem or progenitor cells. In this view, iPS cell technology offers a potential breakthrough, as patient-derived iPS cells can be differentiated toward different cell lineages and yet retain the genetic background of the patients in 2D or 3D cell systems. 3D organoids were developed for a wide range of human tissues or organs for disease modelling and promises to bridge the gap between *in vitro* monolayer cell cultures and *in vivo* studies in animal models (Jiang et al., 2022; Jalal t al. 2021). Furthermore, human organoids are employed to study muscle genetic disorders, including Duchenne muscular dystrophy (DMD). After gene editing corrections, it is possible to obtain isogenic controls to better understand the pathological phenotype and to evaluate the effects of new therapeutic strategies more rigorously, during drug screening or genetic interventions (Marini et al., 2022 now published).

## MABs, cardiac differentiation and organoid technologies

During embryonic development, skeletal muscle precursors arise from paraxial mesoderm (Biressi et al., 2007) while splanchnic, cardiogenic mesoderm gives rise to the heart (Abu-Issa and Kirby et al., 2007) with the participation of neural crest cells. During embryonic development, cardiac or skeletal muscle lineages rely on very specific gene expression networks, often recapitulated during adult myogenesis; the regeneration capacity is very limited in the cardiac tissue compared to skeletal muscles and the presence of cardiac progenitor stem cells is still highly debated (Eschenhagen et al., 2017; Chien et al., 2019; Salerno et al., 2022). Preclinical and clinical studies provided arguments for and against the use of various adult stem cell types for therapeutic approaches, including skeletal myoblasts, bone marrow-derived mononuclear cells mesenchymal stem cells, hematopoietic stem cells, endothelial progenitor cells, and cardiac stem/progenitor cells (Duelen and Sampaolesi. 2017; Eschenhagen et al., 2017; Salerno et al., 2022). In the case of MABs isolated from embryonic dorsal aorta, we observed that intra-ventricular injection was effective in reducing postischemic injury *via* paracrine mechanisms as transplanted cells were unable to differentiate into cardiomyocytes (CMs) (Galli D et al., 2005). Cells with similar features to MABs were isolated from murine (Galvez et al., 2008), human (Galvez et al., 2008), and canine (Cassano et al., 2012) cardiac explants and named cardiac MABs (cMABs). cMABs display pericyte markers together with cardiac transcription factors, differentiate into beating CMs and, after intraventricular delivery in ischemic murine hearts, they contribute directly to the cardiac tissue (Galvez et al., 2009). However, canine, and human MABs showed less proliferative capacity and their cardiac differentiation was modest. Although cMABs may represent a reservoir of cardiac cell progenitors, given the relative abundance of pericytes (from which MABs are derived) around the microvasculature of heart, the fact that they were prone to senescence reduced the enthusiasm for further investigation that was rather directed towards iPS derived cardiac progenitors. Indeed MAB-derived iPS cells show an intrinsic skew towards myogenic commitment, when compared to fibroblast-derived iPS cells and in addition, we could derive from them mesodermal progenitors (MiPs, as iPS cell derived-mesodermal progenitors) able to differentiate in both skeletal and cardiac myocytes, which was unexpected given the different embryonic origin of the 2 cell types (Giacomazzi et al., 2017). MiPs were amenable to gene correction and when systemically injected in dystrophic mice could improve cardiac and skeletal muscle functions. The translational applications of human MiPs for striated muscle repair have not yet been fully addressed but these initial studies are quite promising for a potential therapy to target both cardiac and skeletal dystrophic muscles.

It is largely accepted that murine animal models do not fully resemble human features of DMD degeneration and its disease progression and DMD canine models that provide insight regarding disease pathogenesis and treatment efficacy are expensive and raise many ethical issues. For these reasons, the research of new and more specific treatments aimed to prevent and counteract fibrosis and the resulting cardiomyopathies in DMD patients is hindered. Current advances in organoid technology offered an unprecedented avenue to generate cardiac organoids for studying human heart development *in vitro* that circumvents the limitations of 2D cell culture systems in terms of CM differentiation and maturation (Guilbert et al., 2021). Following this idea, we have generated DMD cardiac organoids that show fibrosis and adipogenesis as typical degenerative features of dystrophic cardiac phenotype upon long-term dynamic culture (up to 93 days), (Marini et al., 2022). These cardiac organoids were generated from both DMD patient-iPS cells, and its isogenic controls corrected *via* CRISPR/Cas9 gene editing technology (Duelen et al., 2022), revealing mitochondrial dysfunction and intracellular ROS concentrations and premature death in DMD iPS cell-CMs. The oxidative stress observed in DMD iPS cell-CMs was mainly due to the overexpression of NOX4 an enzyme family member that contributes to a wide range of pathological processes including, excessive ROS production (Duelen et al., 2022).

Despite exhibiting some clinically relevant pathological signs including, calcium overload, oxidative stress, mitochondrial dysfunction, fibrosis, and aberrant fat replacements, DMD cardiac organoid formation is a spontaneous process of CM differentiation from human iPS cells and exhibits 3D cellular structures controlled mainly by Wnt/BMP signaling factors present in culture medium. Thus, current cardiac organoid technologies have limitations due to cell heterogeneity, low reproducibility in terms of size, and in case of DMD organoids premature cell death due to likely culture conditions that enhance dystrophic CM dysfunctions. These shortcomings hamper the robustness of organoids as accurate 3D human disease cell models for toxicology studies and drug screening. It is therefore mandatory to develop advanced 3D human DMD cellular models that adequately exhibit the human disease phenotypes with high controllability, reproducibility and predictivity at an anatomical spatial resolution. Hereof 3D bioprinting technology is able to develop multi-layered models composed of different cell populations and biomaterials that are printed with a highly controlled cytoarchitecture and very short production cycle times. Thus, cardiac multilayer models containing myocardium and endocardium cell populations including endothelial cells could facilitate neovascularization for better post-transplant engraftments (Kawai et al., 2002).

## MABs for the therapy of muscular dystrophies: How to obtain enough cells

The MAB ability to cross the vessels in the presence of inflammation, made them obvious candidates for systemic cell therapy of muscular dystrophies, and possibly other monogenic disorders of the mesoderm.

We consequently focused on large-scale propagation of cells. The MAB isolation from murine, canine, and human tissue had always been based on a preliminary short-time primary culture of muscle tissue fragments, essential to increase MABs proportion among different cell population outgrowth and allow more efficient selection procedures (Tonlorenzi et al., 2007; Tonlorenzi et al., 2017).

This step was particularly important for human samples, since we could derive MAB cell lines starting from very small skeletal muscles biopsies (sometimes of only 100 mg).

For murine tissues we introduced a mild enzymatic dissociation followed by a cloning step by limited dilution, in presence of a murine feeder layer. Different clones were subsequently screened respect to their proliferation and differentiation efficiency.

Murine MABs could be easily derived and propagated using basic culture media with high FBS content (DMEM supplemented with 20%FBS) in standard culture conditions (5% CO2).

Isolation and propagation of human MABs, in view of clinical use, posed a number of problems, especially regarding propagation and selection.

In most used media and in standard culture conditions human MABs undergo senescence after 10 or at most 20 population doublings (PD). After cloning, life span of derived cell lines was even shorter (less than 10 PD), on top of the fact that the use of a murine feeder layer was not recommendable.

Thanks to the use of “new generation” incubators, we could test low oxygen atmosphere, and found that 5% CO2 and 3–5% O2 tension could mimic more physiologic conditions for MAB cultures.

We than tried to identify a more efficient culture medium and found that Megacell DMEM, supplemented with 5% FBS and 5 ng/ml bFGF supported extensive proliferation, especially under physiological O2 tension, allowing a relevant extension of cells life span.

Under these conditions we could obtain more than a billion cells from a single biopsy. At that stage, cells remained able to differentiate and with a normal karyotype.

More recently we identified Myocult SF as a new serum-free medium that promotes virtually unlimited proliferation of MABs, provided that cells are spared any stress as much as possible (such as introducing the use of a hypoxic station for cell expansion and manipulation).

For what concerns selection, unfortunately MABs, as pericytes derived cells, do not express any unique antigen, except for TNAP whose expression on the surface is not constant so that prospective isolation of MABs is very inefficient.

Our actual selection procedures consist mainly of CD56^+^ myogenic cell fraction depletion by magnetic microbeads separation and verification of standard mesoderm surface markers.

## MABs for the therapy of muscular dystrophies: From bench to bedside

**FIGURE 5:**
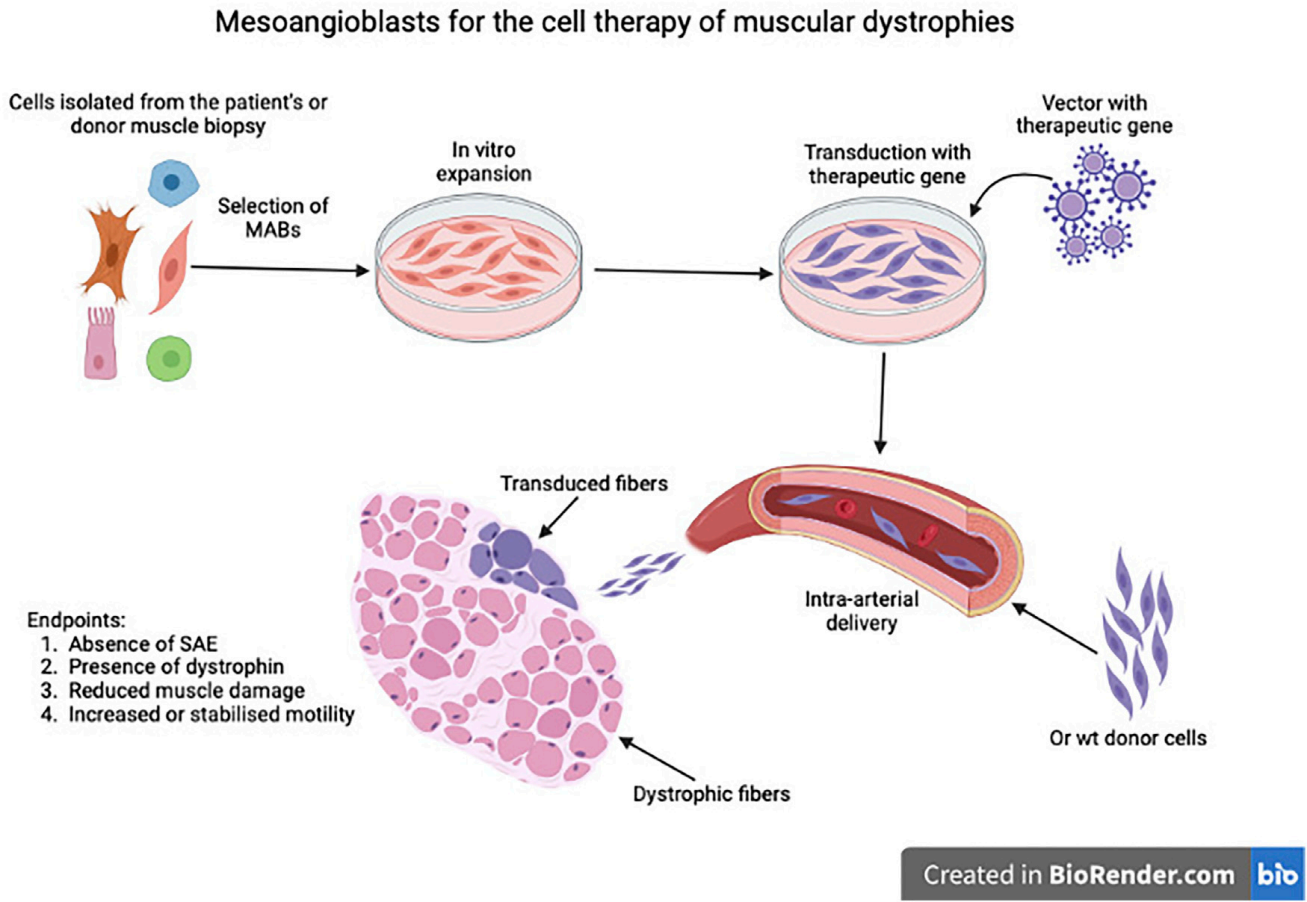
Schematic representation of the process leading to isolation, expansion, eventual transduction or mesoangioblasts as Advanced Therapy Medicinal Product (ATMP) and subsequent intra-arterial administration. SAE: Severe Adverse Event.

While efforts to expand human MABs for future clinical use were ongoing, we carried out of pre-clinical work in three mouse and one dog model (Sampaolesi et al., 2003; Sampaolesi et al., 2006; Diaz-Manera et al., 2010; Tedesco et al., 2011), that showed safety and efficacy of this protocol, schematised in Figure 5.

Results in pre-clinical models were very encouraging with all endpoints of the protocol reached. Both in mice and dogs we observed extensive expression of dystrophin or sarcoglycans or dysferlin, amelioration of the pathology and increased force of contraction, though in no case the levels of heathy animals were reached. Thus, we completed a first in man trial (Cossu et al., 2015) based upon four consecutive intra-femoral arterial administrations of increasing doses of HLA-matched donor MABs (from a sibling) in five DMD patients. We had chosen donor MAB transplantation, based on the superior outcome we observed in dogs, in comparison with autologous cells transduced with a lentivector expressing human micro-dystrophin. The trial showed safety but minimal efficacy, even though we detected, in the youngest patient donor derived dystrophin in the range detected by trials with oligonucleotides for exon skipping (Goemans et al., 2011). Differences with pre-clinical models were mainly due to the advanced age of patients (chosen for safety reasons), the ongoing treatment with steroids (that inhibits MABs adhesion to the endothelium), the lower cell dose and the different posture between human and other mammals, so that targeting leg muscles only is not sufficient to maintain posture and ambulation. The publication of the article reporting the results of the trial was generally interpreted as final evidence that cell therapy would not work for muscular dystrophy, especially with a systemic delivery that would not lead to an engraftment higher than a few percentages of the total population in the best scenario. In contrast, intra-muscular injection of satellite cell-derived progenitors showed some efficacy in localised forms such as OPMD (Oculo-Pharyngeal-Muscular-Dystrophy: Periè et al., 2014) where only pharyngeal muscles were injected.

In the meanwhile, we worked out a way to overcome the unavoidably low engraftment of transplanted cells in tissues where resident, diseased cells cannot be ablated, as in the bone marrow (Cossu et al., 2018).

Various strategies were undertaken to improve MABs engraftment *in vivo* in models of muscle injury and DMD. Some of these strategies exploited signalling molecules that mediate the crosstalk between the immune system and the muscle regenerative niche upon muscle injury.

Proper skeletal muscle regeneration depends on a highly coordinated sequential events as inflammation phases, tissue formation and maturation, that in turn involve the complex orchestration of tissue-resident and recruited cells. Macrophages (MPs) are the major infiltrating population in injured muscle. A simplified view suggests that activated MPs generate M1 (“classically activated”) mainly involved in the response against pathogens and M2 (“alternatively activated”) cells involved in later phases of inflammation, and tissue regeneration, playing also angiogenic and vascular protective roles in inflamed tissues (Arnold et al., 2007; Brunelli and Rovere-Querini, 2008). Factors produced by macrophages can influence skeletal muscle regeneration by stimulating satellite cell survival, proliferation, migration and differentiation (Chazaud et al., 2003; Hara et al., 2011; Chazaud, 2020). It was postulated that the same signals could promote the recruitment and engraftment of transplanted mesangioblast at the site of muscle injury.

One of such factors is nitric oxide (NO). In the skeletal muscle NO is synthesized from l-arginine by NO synthase (NOS) enzymes (Rigamonti et al., 2013). All NOS deficient mice display impaired muscle regeneration in accordance with NO non redundant role in promoting myogenic precursor cell activation and differentiation (De Palma, et al., 2010; Buono, et al., 2012; Rigamonti et al., 2013).

NO donors where therefore administered to mouse models of acute and chronic muscle injury, transplanted with MABs. In agreement with the original hypothesis, it was shown that a brief pre-treatment of with NO donors was sufficient to enhances MAB ability to migrate to the dystrophic muscle, yielding a recovery of its function (Sciorati et al., 2006). In addition, the combination of NO release and nonsteroidal anti-inflammatory drugs significantly improved the engraftment of MABs in the dystrophic muscles by increasing their homing (Brunelli et al., 2007).

Another chemokine generated at the site of tissue injury is High mobility group box 1 (HMGB1). HMGB1 is a highly conserved DNA binding protein, but in addition to its nuclear activities, HMGB1 also plays a role as a signal of tissue damage or a damage-associated molecular pattern when released passively or actively in the extracellular medium (Scaffidi, et al., 2002). HMGB1 was initially explored as a hypothetical chemoattractant for mesangioblasts *in vitro* and *in vivo*. *In vitro* assays revealed that recruitment of MABs required the secretion of HMGB1 and TNF-α by M1 macrophage (Lolmede et al., 2009). *In vivo*, it was shown that exogenous HMGB1 is sufficient to attract MABs when injected in healthy muscle, and that it improved their migration in the dystrophic muscle in a RAGE independent way (Palumbo et al., 2004). This is indeed in accordance with recent work that demonstrated that in the regenerating skeletal muscle the activity of HMGB1 is dependent on its redox state: HMGB1 exerts its chemoattractive action only when in a reduced state, *via* the CXCR4 receptor, while the oxidized disulfide HMGB1 acts as a proinflammatory cytokine signalling through receptors TLR4/MD-2 and RAGE (Venereau et al., 2012; Tirone et al., 2018).

We also studied adhesion of MABs to the endothelium, aiming at implementing trans-endothelial migration and thus enhance cell engraftment. We observed that steroids reduce expression of endothelium adhesion molecules and it is sufficient to remove steroids for a day to increase up to eightfold MAB adhesion *in vitro*. Interestingly steroids do not cause downregulation of endothelial adhesion molecules such as VCAM but their sialylation that prevents cell binding (Meggiolaro et al. in preparation). In addition, a recent report (Ku et al., 2017) showed that Pioglitazone and Rosiglitazone inhibit endothelial expression of JAMA (Junctional Adhesion Molecule A). Since we had previously shown that silencing JAMA would reduce MAB extravasation (Giannotta et al., 2014 EMBO Mol. Med. 2014) and considering that Pioglitazone is used in paediatric clinical trials, that would be an ideal candidate for a combinatorial therapy.

Even if all the above strategies enhance MAB engraftment in murine models of muscle dystrophy, the extent of engraftment in human muscle would remain so low that improving engraftment alone would not lead to clinical efficacy.

**FIGURE 6:**
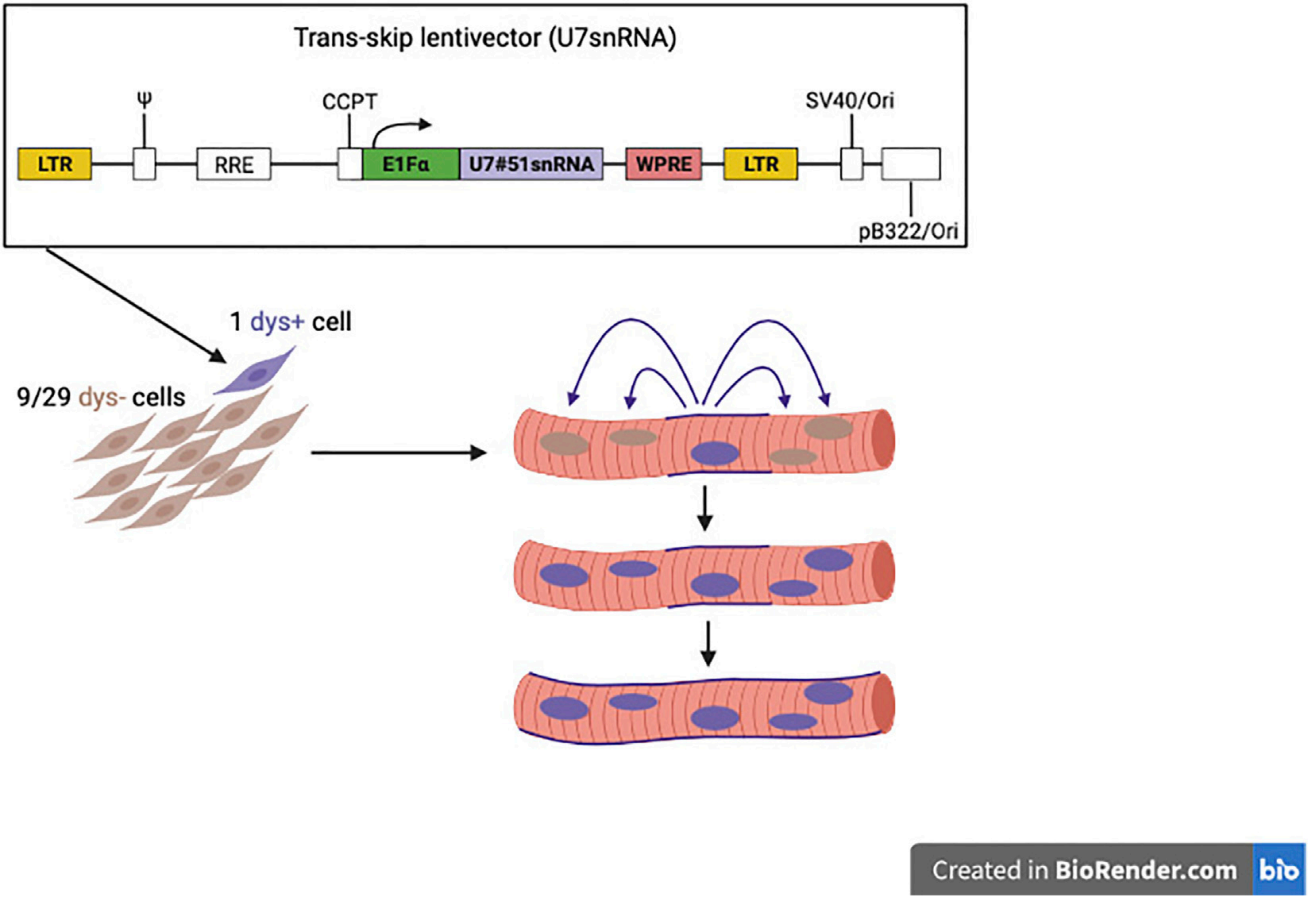
Novel strategy to reach quasi-normal expression of dystrophin, with donor cells working as “trojan horses” since the few nuclei penetrated inside the host fibre correct also the resident nuclei, like the few Trojan warriors penetrated inside the horse donated by the Greeks, surprised the Troy residents during the sleep and managed to conquered the city.

Thus, we completed a study using a new strategy of genetic correction that compensates for the poor engraftment of donor cells in skeletal muscle. This is a cell-mediated exon skipping. DMD MABs are transduced with a lentivector expressing a snRNA engineered to skip exon 51 (De Angelis et al., 2002). Since the snRNA is produced by a donor nucleus, assembles in the cytoplasm and then enters all the neighbouring nuclei, this mechanism amplifies several folds the production of dystrophin both *in vitro* and in immune deficient DMD mice with a skippable mutation of exon 51 (Galli et al. in preparation) resulting in a much higher production of dystrophin than when healthy cells, that can only produce their own dystrophin, are injected. This is schematically shown in Figure 6.

We are testing safety of genetically corrected (with the strategy described above) autologous MABs by intramuscular injection in a foot muscle of five non-ambulant DMD patients. In case of dystrophin production ≥10% of a healthy muscle, cells will also be injected in the thumb muscle, whose increased force of contraction would ameliorate the quality of patients’ life. The trial started in Manchester in the fall 2018 but was halted by COVID and the closure of the GMP facility in Manchester and has now restarted utilising a new GMP facility in Switzerland and the San Raffaele Hospital as clinical centre.
