# Correction: Mesoangioblasts at 20: from the embryonic aorta to the patient bed

**DOI:** 10.3389/fgene.2026.1884212

**Published:** 2026-07-08

**Authors:** Giulio Cossu, Rossana Tonlorenzi, Silvia Brunelli, Maurilio Sampaolesi, Graziella Messina, Emanuele Azzoni, Sara Benedetti, Stefano Biressi, Chiara Bonfanti, Laricia Bragg, Jordi Camps, Ornella Cappellari, Marco Cassano, Fabio Ciceri, Marcello Coletta, Diego Covarello, Stefania Crippa, M. Gabriella Cusella-De Angelis, Luciana De Angelis, Arianna Dellavalle, Jordi Diaz-Manera, Irene Fancello, Daniela Galli, Francesco Galli, Cesare Gargioli, Mattia F. M. Gerli, Giorgia Giacomazzi, Beatriz G. Galvez, Hidetoshi Hoshiya, Maria Guttinger, Anna Innocenzi, M. Giulia Minasi, Laura Perani, Stefano C. Previtali, Mattia Quattrocelli, Martina Ragazzi, Urmas Roostalu, Giuliana Rossi, Sabrina Santoleri, Raffaella Scardigli, Dario Sirabella, Francesco Saverio Tedesco, Yvan Torrente, Gonzalo Ugarte

**Affiliations:** 1 Division of Cell Matrix Biology and Regenerative Medicine. University of Manchester, Manchester, United Kingdom; 2 Division of Neuroscience, IRCCS Ospedale San Raffaele, Milan, Italy; 3 Muscle Research Unit, Charité Medical Faculty and Max Delbrück Center, Berlin, Germany; 4 School of Medicine and Surgery, University of Milano Bicocca, Milan, Italy; 5 Translational Cardiomyology Laboratory, Stem Cell and Developmental Biology Unit, Department of Development and Regeneration, KU Leuven, Leuven, Belgium; 6 Histology and Medical Embryology Unit, Department of Anatomy, Forensic Medicine and Orthopaedics, Sapienza University, Rome, Italy; 7 Department of Biosciences, University of Milan, Milan, Italy; 8 UCL Great Ormond Street Institute of Child Health and NIHR GOSH Biomedical Research Centre, London, United Kingdom; 9 Department of Cellular, Computational and Integrative Biology (CIBIO) and Dulbecco Telethon Institute, University of Trento, Trento, Italy; 10 Bayer AG, Research and Development, Pharmaceuticals, Berlin, Germany; 11 Department of Pharmacy-Drug Sciences, University of Bari “Aldo Moro”, Bari, Italy; 12 Lunaphore Technologies SA, Tolochenaz, Switzerland; 13 AGC Biologicals, Milan, Italy; 14 San Raffaele-Telethon Institute of Gene Theray, IRCCS Ospedale SanRaffaele, Milan, Italy; 15 Department of Anatomy, University of Pavia, Pavia, Italy; 16 Cord Blood Bank, InScientiaFides, San Marino; 17 John Walton Muscular Dystrophy Research Centre, Newcastle University, United Kingdom; 18 Department of Medicine and Surgery, University of Parma, Parma, Italy; 19 Department of Biology, University of Tor Vergata, Rome, Italy; 20 UCL Department of Surgical Biotechnology and Great Ormond Street Institute of Child Health, London, United Kingdom; 21 CellCarta, Gosselies, Belgium; 22 Department of Biochemistry and Molecular Biology, Faculty of Pharmacy, Universidad Complutense de Madrid, Madrid, Spain; 23 CellFiber Co., Ltd., Tokyo, Japan; 24 IFO, Istituti Fisioterapici Ospedalieri, Rome, Italy; 25 Lavitaminasi, Clinical Nutrition and Reproductive Medicine, Rome, Italy; 26 Division of Molecular Cardiovascular Biology, University of Cincinnati, Cincinnati, OH, United States; 27 Telethon Foundation, Milan, Italy; 28 Gubra ApS, Horsholm, Denmark; 29 Roche Institute for Translational Bioengineering (ITB), pRED Basel, Basel, Switzerland; 30 Institute of Translational Pharmacology, National Research Council, Rome, Italy; 31 Columbia Stem Cell Initiative, Departmentof Rehabilitation and Regenerative Medicine, Columbia University, New York, United States; 32 University College London, Great Ormond Street Hospital for Children and The Francis Crick Institute, London, United Kingdom; 33 Neurology Unit, Fondazione IRCCS Ca’ Granda Ospedale Maggiore Policlinico, Milan, Italy; 34 Stem Cell Laboratory, Department of Pathophysiology and Transplantation, Dino Ferrari Center, University of Milan, Milan, Italy; 35 Laboratory of Neuroscience, Faculty of Chemistry and Biology, University of Santiago de Chile, Santiago, Chile

**Keywords:** mesoderm, myogenic stem cells, pericyte, muscular dystrophy, muscle development

Author Irene Fancello and Sabrina Santoleri were omitted as authors in the published article. The following authors' affiliations were erroneously assigned. The incorrect affiliations have now been removed for these authors. The correct affiliations are as follows: Martina Ragazzi: Telethon Foundation, Milan, Italy; Urmas Roostalu: Gubra ApS, Horsholm, Denmark; Giuliana Rossi: Roche Institute for Translational Bioengineering (ITB), pRED Basel, Basel, Switzerland; Raffaella Scardigli: Institute of Translational Pharmacology, National Research Council, Rome, Italy; Dario Sirabella: Columbia Stem Cell Initiative, Department of Rehabilitation and Regenerative Medicine, Columbia University, New York, United States; Francesco Saverio Tedesco: University College London, Great Ormond Street Hospital for Children and The Francis Crick Institute, London, United Kingdom; Yvan Torrente: Neurology Unit, Fondazione IRCCS Ca' Granda Ospedale Maggiore Policlinico, Milan; Stem Cell Laboratory, Department of Pathophysiology and Transplantation, Dino Ferrari Center, University of Milan; Gonzalo Ugarte: Laboratory of Neuroscience, Faculty of Chemistry and Biology, University of Santiago de Chile, Santiago, Chile.

The correct author list reads:

Giulio Cossu^1,2,3^*, Rossana Tonlorenzi^2^*, Silvia Brunelli^4^*, Maurilio Sampaolesi^5,6^*, Graziella Messina^7^*, Emanuele Azzoni^4^, Sara Benedetti^8^, Stefano Biressi^9^, Chiara Bonfanti^7^, Laricia Bragg^1^, Jordi Camps^10^, Ornella Cappellari^11^, Marco Cassano^12^, Fabio Ciceri^2^, Marcello Coletta^6^, Diego Covarello^13^, Stefania Crippa^14^, M. Gabriella Cusella-De Angelis^15^, Luciana De Angelis^6^, Arianna Dellavalle^16^, Jordi Diaz-Manera^17^, Irene Fancello^1^, Daniela Galli18, Francesco Galli^1^, Cesare Gargioli^19^, Mattia F. M. Gerli^20^, Giorgia Giacomazzi^21^, Beatriz G. Galvez^22^, Hidetoshi Hoshiya^23^, Maria Guttinger^24^, Anna Innocenzi^2^, M. Giulia Minasi^25^, Laura Perani^2^, Stefano C. Previtali^2^, Mattia Quattrocelli^26^, Martina Ragazzi^27^, Urmas Roostalu^28^, Giuliana Rossi^29^, Sabrina Santoleri^1^, Raffaella Scardigli^30^, Dario Sirabella^31^, Francesco Saverio Tedesco^32^, Yvan Torrente^33,34^ and Gonzalo Ugarte^35^

1Division of Cell Matrix Biology and Regenerative Medicine. University of Manchester, Manchester, United Kingdom, 2Division of Neuroscience, IRCCS Ospedale San Raffaele, Milan, Italy, 3Muscle Research Unit, Charité Medical Faculty and Max Delbrück Center, Berlin, Germany, 4School of Medicine and Surgery, University of Milano Bicocca, Milan, Italy, 5Translational Cardiomyology Laboratory, Stem Cell and Developmental Biology Unit, Department of Development and Regeneration, KU Leuven, Leuven, Belgium, 6Histology and Medical Embryology Unit, Department of Anatomy, Forensic Medicine and Orthopaedics, Sapienza University, Rome, Italy, 7Department of Biosciences, University of Milan, Milan, Italy, 8UCL Great Ormond Street Institute of Child Health and NIHR GOSH Biomedical Research Centre, London, United Kingdom, 9Department of Cellular, Computational and Integrative Biology (CIBIO) and Dulbecco Telethon Institute, University of Trento, Trento, Italy, 10Bayer AG, Research and Development, Pharmaceuticals, Berlin, Germany, 11Department of Pharmacy-Drug Sciences, University of Bari “Aldo Moro”, Bari, Italy, 12Lunaphore Technologies SA, Tolochenaz, Switzerland, 13AGC Biologicals, Milan, Italy, 14San Raffaele-Telethon Institute of Gene Theray, IRCCS Ospedale SanRaffaele, Milan, Italy, 15Department of Anatomy, University of Pavia, Pavia, Italy, 16Cord Blood Bank, InScientiaFides, San Marino, 17John Walton Muscular Dystrophy Research Centre, Newcastle University, United Kingdom, 18Department of Medicine and Surgery, University of Parma, Parma, Italy, 19Department of Biology, University of Tor Vergata, Rome, Italy, 20UCL Department of Surgical Biotechnology and Great Ormond Street Institute of Child Health, London, United Kingdom, 21CellCarta, Gosselies, Belgium, 22Department of Biochemistry and Molecular Biology, Faculty of Pharmacy, Universidad Complutense de Madrid, Madrid, Spain, 23CellFiber Co., Ltd., Tokyo, Japan, 24IFO, Istituti Fisioterapici Ospedalieri, Rome, Italy, 25Lavitaminasi, Clinical Nutrition and Reproductive Medicine, Rome, Italy, 26Division of Molecular Cardiovascular Biology, University of Cincinnati, Cincinnati, OH, United States, 27Telethon Foundation, Milan, Italy, 28Gubra ApS, Horsholm, Denmark, 29Roche Institute for Translational Bioengineering (ITB), pRED Basel, Basel, Switzerland, 30Institute of Translational Pharmacology, National Research Council, Rome, Italy, 31Columbia Stem Cell Initiative, Departmentof Rehabilitation and Regenerative Medicine, Columbia University, New York, United States, 32University College London, Great Ormond Street Hospital for Children and The Francis Crick Institute, London, United Kingdom, 33Neurology Unit, Fondazione IRCCS Ca' Granda Ospedale Maggiore Policlinico, Milan, Italy, 34Stem Cell Laboratory, Department of Pathophysiology and Transplantation, Dino Ferrari Center, University of Milan, Milan, Italy, 35Laboratory of Neuroscience, Faculty of Chemistry and Biology, University of Santiago de Chile, Santiago, Chile.

The Author Contributions Statement has been corrected to read:

During these 20 years, all the authors contributed to the work. LDA, MGM, MC, RT and LB developed the methods to isolate and culture mesoangioblasts. FC, SCP and YT were responsible for the clinical translation, all other authors studied the biology of these cells both *in vitro* and after transplantation in animal models. GC, RT, SB, MS and GM wrote the manuscript. SB and GM drew the figures. IF and SS engaged in data curation, formal analysis, investigation, and writing – review and editing.

There was a typo in the conflict of interest statement. “Gupra” has been corrected to “Gubra”.

The original version of this article has been updated.

